# Higher Incidences of Severe Medical Emergencies and Poorer Out-of-Hospital Cardiac Arrest Outcomes in Farmlands Compared to Other Outdoor Workplaces

**DOI:** 10.7759/cureus.76838

**Published:** 2025-01-03

**Authors:** Koichi Tanaka, Ayako Haraguchi, Takashi Iwasaki, Hideo Inaba

**Affiliations:** 1 Department of Emergency Medical Science, Niigata University of Health and Welfare, Niigata, JPN; 2 Department of Social Welfare, Niigata University of Health and Welfare, Niigata, JPN; 3 Department of Emergency Medicine, Kanazawa Medical University, Uchinada, JPN

**Keywords:** agricultural land, bystander cardiopulmonary resuscitation, detection of emergencies, emergency medical service, outcome, out-of-hospital cardiac arrest, outpatient death, public access defibrillation, response time interval, rurality

## Abstract

Introduction: Farmland is an essential yet hazardous workplace where an aging population is engaged. This study aimed to compare the characteristics and severity of emergencies between farmlands and other outdoor workplaces and clarify whether the rurality might influence the differences between farmlands and other outdoor workplaces.

Methods: This retrospective cohort study analyzed the nationwide emergency medical service (EMS) transportation database between 2016 and 2021, combined with out-of-hospital cardiac arrest (OHCA) data.

Findings: Farmlands emergencies accounted for 0.26% of all non-pediatric (≥15 years) emergencies (72,162 out of 27,998,839) and 40.6% of outdoor workplace emergencies (177,923 cases). These emergencies were less frequent in winter (12.9% versus 19.7%) and during daytime hours (83.3% versus 87%) but more common in rural areas (31.4% versus 15.7%) compared to other outdoor workplaces. Farmland emergencies involved a lower proportion of male patients (67.6% versus 94.1%) and a higher proportion of older adults (≥60 years) (85.9% versus 34%), medical emergencies (42.7% versus 33.8%), outpatient deaths (3.9% versus 1.1%), and out-of-hospital cardiac arrests (OHCA) (5.1% versus 2.7%). EMS response and transport times were significantly longer for farmland emergencies. These differences in characteristics were more pronounced in non-rural EMS areas. Farmlands were strongly associated with higher outpatient death rates even after adjustment for other factors, with this association further strengthened in specific subgroups: non-rural emergencies, daytime hours, females, and cases not transported to high-class emergency hospitals. Regardless of the EMS rurality, OHCA in farmlands exhibited lower bystander CPR rates, fewer shockable rhythms, and limited public access defibrillation compared to other outdoor workplaces, alongside higher proportions of unwitnessed and medical cases. Neurologically favorable one-month survival was significantly lower in farmlands (1.6% versus 5.4% in rural areas and 2.9% versus 10% in non-rural areas). However, after adjusting for OHCA characteristics, survival differences were not statistically significant (95% confidence interval (CI) of adjusted odds ratio (OR): 0.41-2.63 in rural and 0.52-1.10 in non-rural EMS).

Conclusions: Severe medical emergencies are more common in farmlands, and EMS and bystander responses to OHCA are poorer than in other outdoor workplaces, leading to worse outcomes in OHCA in farmlands. Implementing effective health and safety protocols and strategies to improve preventative health management programs and strengthening collaboration between local EMS and agricultural communities are critical to improving outcomes and aligning with the Sustainable Development Goals (SDGs) for agriculture.

## Introduction

Farmland work, while essential to food production and rural economies [1], is associated with various risks due to the workforce's unique working environment and demographic characteristics. However, farmland is also known to be hazardous, given the risk of various types of accidents, work-related injuries, and health disturbances [2]. Previous guidelines and literature on agricultural safety have primarily addressed work-related chemical injuries and trauma [2,3] because farming involves various tasks, including planting, harvesting, animal care, and machinery operation. Farmers' health is linked to agricultural production efficiency [4] and presumably abandoned farmland [5].

Severe emergencies in farmland may lead to out-of-hospital cardiac arrest (OHCA) if the detection of emergencies and activation of emergency medical services (EMSs) are delayed [6]. Furthermore, OHCA outcomes may deteriorate in the absence of early recognition, bystander-initiated high-quality cardiopulmonary resuscitation (CPR), and early defibrillation [7]. Previous studies on out-of-hospital cardiac arrest (OHCA) outcomes have shown better outcomes in workplaces compared to other locations, likely due to the availability of automated external defibrillators (AEDs) and trained personnel [8-10]. However, these studies excluded farmland from the workplace. Unlike other outdoor locations, farmlands tend to be remote, with uneven terrain that complicates emergency response [2,11]. In addition, agricultural work is heavily influenced by weather and seasonal cycles and often lacks the stringent safety protocols found in other outdoor workplaces [2].

According to data from the Food and Agriculture Organization of the United Nations [12], the global population of agricultural workers was approximately 900 million in 2019, a decrease from approximately one billion in 2000. Mechanization and automation in commercial agriculture have reduced the number of people employed in the agricultural sector [12]. Small family farms still account for a large percentage of the world's farmers, and approximately 75% of the world's farmland is family-owned [12]. As a result, farmlands often lack the stringent occupational health and safety protocols seen in other outdoor workplaces, and preparedness for and actual management of emergencies might be less organized and poorer in farmlands than in other workplaces.

The aging of the agricultural population is a major concern globally [13]. Asia, particularly Japan, faces a rapidly aging farming population [14,15], which may be associated with accelerated farmland abandonment and reduced productivity. The aging farmer population may increase the likelihood of non-accidental, serious medical emergencies occurring on farmlands, as the risk of cardiovascular disease in male farmers over the age of 45 is reported to be high [16]. Furthermore, bystander response to serious emergencies or OHCA in farmlands may be compromised due to an age-dependent decline in knowledge and self-confidence in providing basic life support and cardiopulmonary resuscitation (CPR) [17].

In Japan, 70% of farmers are over 65 years of age [15], which increases the likelihood of medical emergencies and out-of-hospital cardiac arrest (OHCA) in agricultural areas, where emergency medical service (EMS) response may be delayed. Bystander CPR and access to automated external defibrillators (AEDs) are thought to be low in these regions due to limited training and AED availability [2,14,17]. Despite the importance of this issue, no nationwide surveys have been conducted.

In Japan, healthcare costs are generally covered by public insurance and funding. Almost all emergencies are managed by an ambulance team of at least three EMS personnel, including one paramedic. In cases of OHCA, EMS teams cannot terminate resuscitation in the field unless specific conditions, including postmortem changes, are met. As reported previously, paramedics can perform various advanced resuscitation procedures for patients with OHCA [18].

The Fire and Disaster Management Agency (FDMA) recently provided a comprehensive database of EMS-transported emergency cases, including detailed information on location, patient management, and transportation. Using this database, this study aimed to investigate the epidemiology, characteristics, and severity of emergencies in farmlands and agricultural areas compared with those in other outdoor workplaces and clarify whether the rurality of EMS might influence the difference in emergencies between farmland and other outdoor workplaces using subgroup analyses.

## Materials and methods

Ethical consideration

Our local Ethics Committee at Kanazawa Medical University approved this study as a study project (approval number: I-729). The requirement for written informed consent was waived because anonymized secondary data were analyzed.

Study design

This retrospective cohort study analyzed nationwide data on EMS transportation in Japan from January 1, 2016, to December 31, 2021. Data from the All-Japan Utstein-style [19] registry for out-of-hospital cardiac arrest (OHCA) was used to identify cases of resuscitation-attempted OHCA and their outcomes. This study was conducted following the Strengthening the Reporting of Observational Studies in Epidemiology guidelines for observational studies.

Databases and participants

All fire departments in Japan are involved in collecting data for the nationwide EMS transport database. FDMA cleaned the data collected from the fire departments and managed the quality of the database by requesting the fire departments to revise logically incorrect data. From these transport data in Japan, we extracted non-pediatric emergency cases transported by EMS from outdoor workplaces because the Labor Standards Law in Japan prohibits work by persons under 15 years of age. The FDMA database categorizes workplaces into farmlands/agricultural areas, industry/factories, business/industrial offices, indoor and outdoor non-industrial work areas, indoor and outdoor construction sites, warehouses, underground spaces, hangars, welfare facilities, corridors, and bathrooms. We have extracted emergency cases from farmlands/agricultural areas, outdoor construction sites, and work areas as outdoor workplaces.

Sample size calculation

Statistical sample size calculations were not performed before this large, population-based observational study because there were no similar studies of emergencies and OHCA in agricultural settings. The sample size was determined by the number of emergencies and OHCA cases transported by EMS during the study period. This decision was based on the retrospective nature of the study and the feasibility of collecting all eligible cases within the defined time frame. To ensure the robustness of the results, post hoc power calculations were performed for the primary outcome in each analysis using a chi-squared test with continuity correction and a two-sided significance level of P < 0.05 [20]. The observed power levels were greater than 99% for the primary and secondary outcomes, indicating sufficient statistical confidence.

EMS subgroup

Based on the Law on Special Measures to Promote the Independence of Underpopulated Areas [21], the Ministry of the Interior and Communications will designate 713 rural municipalities (cities and towns) in 2022 according to population and financial strength criteria.

Population requirements must meet one of the following criteria: the rate of population decline between 1960 and 1995 ≥ 30%, the rate of population decline between 1960 and 1995 ≥ 25%, and the proportion of the population aged ≥65 ≥ 25% or the proportion of the population aged 15-29 ≤ 25%. Financial requirements must meet the average fiscal capacity index from fiscal year (FY) 1996 to 1998 ≤ 0.42, and revenue from publicly operated games ≤ 1.3 billion yen.

Rural fire departments were defined as those where more than 50% of the population covered by EMS lived in municipalities in depopulated areas.

Outcome measures

The primary outcome was the severity of emergencies, categorized as outpatient discharge, hospital admission, and outpatient death. Unlike general criteria, outpatient death was defined as a declaration of death in emergency departments or before hospital admission. The secondary outcome of this study was neurologically favorable one-month survival, defined as Cerebral Performance category [22] 1 or 2 in the OHCA cohort.

Statistical analysis

Differences in variables between farmland/agricultural areas and other outdoor workplaces were first analyzed using the chi-squared test for categorical variables and the Mann-Whitney U test for continuous variables in all tables. When the P value of the chi-squared test was <0.05, we reported the crude odds ratios (ORs) and 95% confidence intervals (CIs) with other outdoor workplaces as a reference. Subgroup analyses using multivariate interaction tests including all variables in tables assessed whether the EMS rurality might modify the effect of farmland/agricultural areas on each variable. Multivariate logistic regression with interaction test was applied to determine the factors associated with outpatient death, followed by subgroup analyses. Comparisons of neurologically favorable one-month survival between farmland/agricultural areas and other outdoor workplaces using univariate analyses were followed by adjustment using multivariate logistic regression analyses, including factors known to be associated with outcomes [19]. The null hypothesis was tested in each analysis at a significance level of P < 0.05. All statistical analyses were performed using JMP software (JMP Pro 17, SAS Institute, Cary, NC).

## Results

Study cohorts and incidences of emergencies in outdoor workplaces

**Figure 1:**
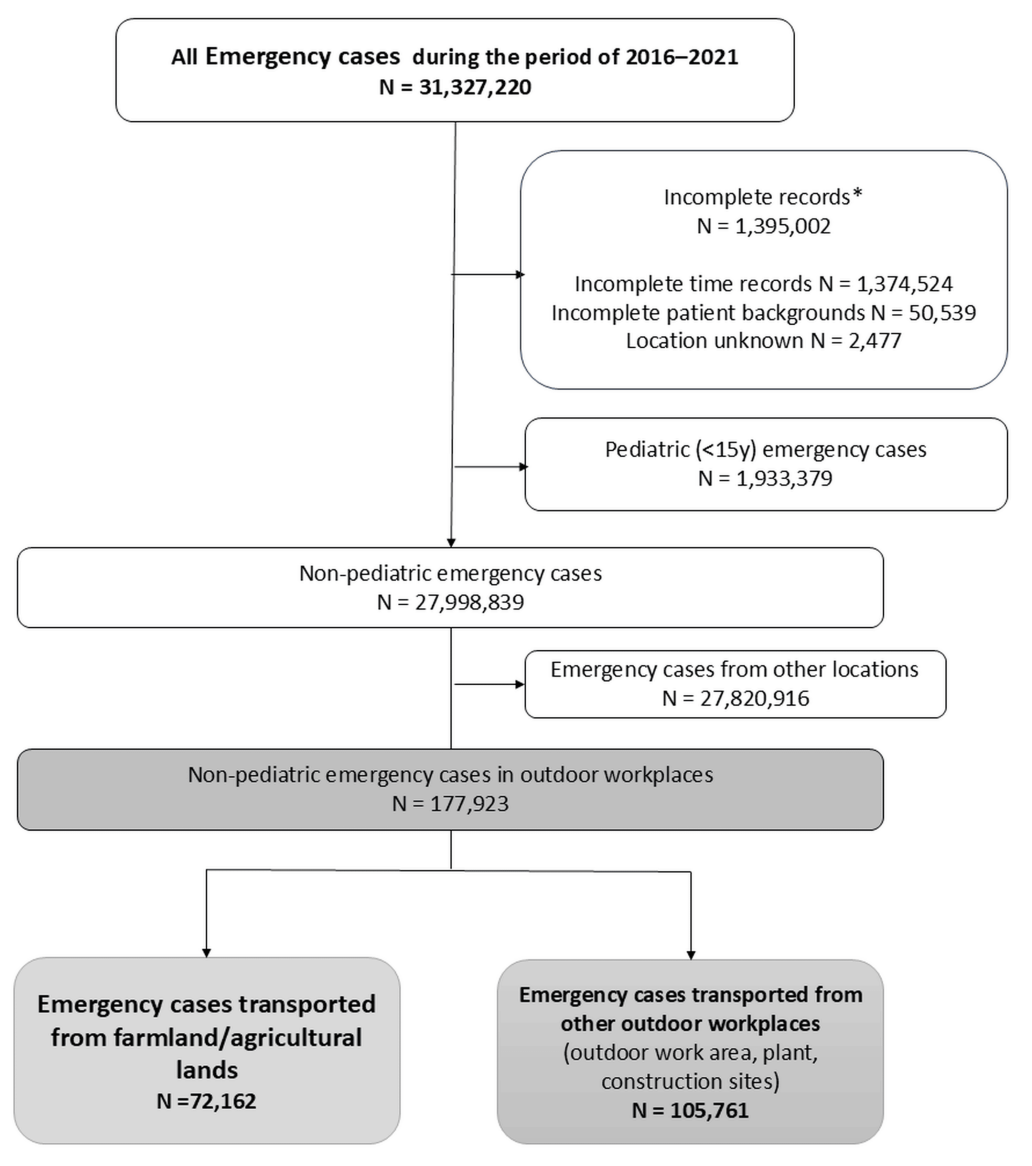
Data selection and overview of emergencies Pediatric emergency cases were excluded because the Labor Standards Law in Japan prohibits people under 15 years of age from working. *The total number of cases with incomplete records is not equal to the sum of the number of cases in three categories because some cases had incomplete records in multiple categories.

Because this study focused on the characteristics and background of outdoor workplace emergencies and EMS time intervals, we first excluded 1,395,002 (4.5%) cases with incomplete records (1,374,524 cases with incomplete time records, 50,539 cases with missing patient background, and 2,477 cases with unknown location) from the 31,327,220 emergency cases transported between 2016 and 2021. Notably, the total number of incomplete cases is not equal to the sum of the number of cases with three exclusion categories because some cases had incomplete records in multiple categories. The Labor Standards Law in Japan prohibits people under the age of 15 from working. Therefore, we excluded 1,933,379 (6.2%) pediatric cases (<15 years) and created the primary data for 27,998,839 (89.3%) non-pediatric emergency cases transported by EMS. From this primary dataset, 177,923 cases transported from outdoor workplaces (72,162 cases from farmland/agricultural areas and 105,761 cases from other outdoor workplaces) were extracted and analyzed for epidemiology, characteristics, and severity of emergencies (Figure 1).

Identification of OHCA cases and OHCA cohort

**Figure 2:**
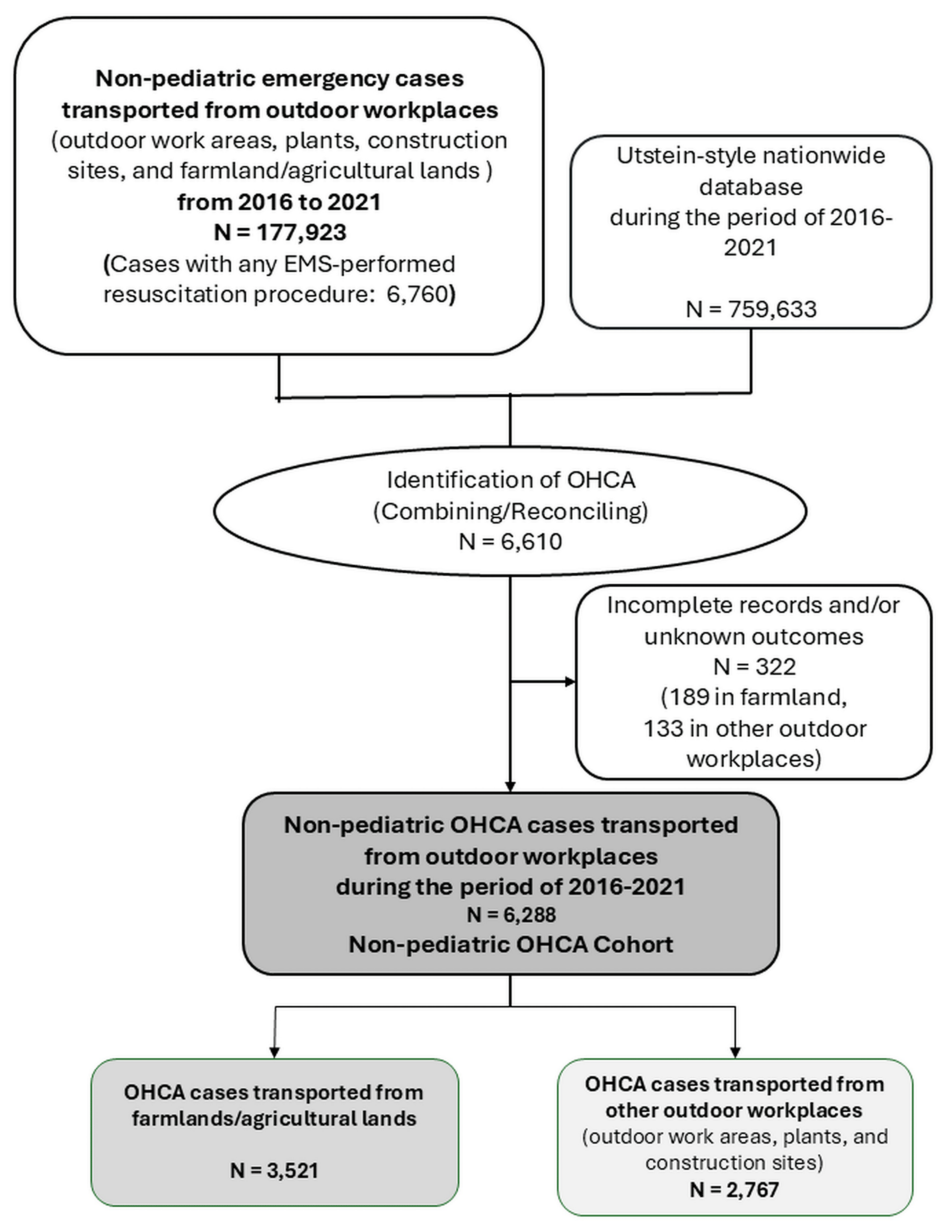
Identification of OHCA cases and data selection for non-pediatric OHCA cases OHCA: out-of-hospital cardiac arrest, EMS: emergency medical service

To identify OHCA cases, we combined the 177,923 non-pediatric emergency cases transported from outdoor workplaces with the Utstein-style nationwide database of 759,633 cases by matching age, sex, prefecture, and time records. This process identified 6,610 cases of OHCA. After excluding 322 cases with incomplete OHCA records and/or unknown outcomes, a secondary dataset of 6,288 OHCA cases with attempted resuscitation in outdoor workplaces (2,767 cases in farmland/agricultural areas) was created to analyze the characteristics and outcomes of OHCA (Figure 2).

The proportions of non-pediatric emergencies in farmland/agricultural areas and other outdoor workplaces were 0.26% and 0.38%, respectively, and the proportions of OHCA in non-pediatric emergencies were 5.1% and 2.7%, respectively.

Epidemiology and characteristics of EMS-transported emergency cases

The number of emergencies in farmland/agricultural areas gradually decreased during the study period. The association between winter and lower incidence of emergencies was pronounced in farmland/agricultural areas (crude OR: 0.61, 95% CI: 0.50-0.62). Compared with other outdoor workplaces, the emergencies in farmland/agricultural areas were characterized by lower incidences in daytime (crude OR: 0.75, 95% CI: 0.73-0.77) and male patients (crude OR: 0.13, 95% CI: 0.12-0.13) and much higher proportions of older patients (≥75 years) (crude OR: 19.8, 95% CI: 19.3-20.5) and rural EMS (crude OR: 2.45, 95% CI: 2.40-2.51). The proportion of medical emergencies (crude OR: 1.46, 95% CI: 1.43-1.49) and cardiovascular events (crude OR: 1.53, 95% CI: 1.48-1.58), including stroke, was higher in farmlands than in other outdoor workplaces (Table 1).

**Table 1:** Comparisons of epidemiology and characteristics of EMS-transported emergency cases between farmland/agricultural lands and other outdoor workplaces OR: odds ratio, CI: confidential interval, EMS: emergency medical service ^a^Crude OR (95% CI) is shown when the chi-squared test detected a significant difference. ^b^Classified based on the list of rural municipalities defined by the Ministry of Internal Affairs and Communication [19]. ^c^Water, traffic, labor, fire accidents, and general injuries.

Epidemiological characters	Farmland/agricultural lands (N = 72,162)	Other outdoor workplaces (N = 105,761)	Crude OR (95% CI) for farmland (all other outdoor workplaces = 1)^a ^or P value
Year, number (%)			P < 0.001
2016/2017	25,405 (35.2%)	34,834 (32.9%)	1.11 (1.08-1.13)
2018/2019	24,096 (33.4%)	36,595 (34.6%)	0.95 (0.93-0.97)
2020/2021	22,661 (31.4%)	34,332 (32.5%)	0.95 (0.93-0.97)
Season, number (%)			P < 0.001
Winter	9,327 (12.9%)	20,817 (19.7%)	0.61 (0.59-0.62)
Spring	17,997 (24.9%)	21,673 (20.5%)	1.29 (1.26-1.32)
Summer	25,445 (35.3%)	38,048 (36.0%)	0.97 (0.95-0.99)
Autumn	19,393 (26.9%)	25,223 (23.9%)	1.17 (1.15-1.20)
Daytime (8:00-17:59), number (%)	60,115 (83.3%)	92,010 (87.0%)	0.75 (0.73-0.77)
Rural EMS^b^, number (%)	22,624 (31.4%)	16,603 (15.7%)	2.45 (2.40-2.51)
Age group, number (%)			P < 0.001
15-39 years	3,040 (4.2%)	27,198 (25.7%)	0.13 (0.12-0.13)
40-59 years	7,154 (9.9%)	42,661 (40.3%)	0.16 (0.16-0.17)
60-74 years	23,680 (32.8%)	30,210 (28.6%)	1.22 (1.20-1.25)
≥75 years	38,288 (53.1%)	5,692 (5.4%)	19.8 (19.3-20.5)
Male, number (%)	48,760 (67.6%)	99,553 (94.1%)	0.13 (0.12-0.13)
Scene classification, number (%)			P < 0.001
Medical	30,050 (41.6%)	34,345 (33.5%)	1.46 (1.43-1.49)
Accidental^c^	40,561 (56.2%)	69,168 (65.4%)	0.68 (0.66-0.69)
Other	773 (1.1%)	856 (0.8%)	1.33 (1.20-1.46)
Cardiovascular diseases, number (%)	8,919 (12.4%)	8,936 (8.5%)	1.53 (1.48-1.58)
Ischemic heart disease	490 (0.7%)	536 (0.5%)	1.34 (1.19-1.52)
Stroke	5,327 (7.4%)	4,607 (4.4%)	1.75 (1.68-1.82)

The EMS time intervals, including response, scene, and transportation times, were longer for farmland/agricultural areas than other outdoor workplaces (P < 0.001). Furthermore, the delays in EMS vehicle dispatch after emergency call receipt and EMS contact with patients after EMS vehicle arrival near the scene were more common in farmlands than in other workplaces (Table 2).

**Table 2:** Comparisons of EMS time intervals between farmland and other outdoor workplaces OR: odds ratio, CI: confidential interval, EMS: emergency medical service, IQR: interquartile range ^a^P value by the Wilcoxon/Kruskal-Wallis test is shown for continuous variables. ^b^Interval between emergency call receipt and EMS contact with patients. ^c^Interval between EMS contact with patients and patient accommodation in the ambulance. ^d^Interval between patient accommodation in the ambulance and that in the hospital.

EMS time interval, minutes, median (IQR)	Out-of-office workplaces		P value^a^ or OR (95% CI) for farmland (all other outdoor workplaces = 1)
	Farmland (N = 72,162)	Other outdoor workplaces (N = 105,761)	
Response time^b^	11 (9-15)	10 (8-13)	P < 0.001
Call receipt to dispatch > 2 minutes, number (%)	18,020 (25.0%)	22,703 (21.5%)	1.22 (0.19-1.25)
Vehicle arrival to contact > 2 minutes, number (%)	10,915 (15.1%)	9,095 (8.6%)	1.89 (1.84-1.95)
Scene time^c^	5 (3-8)	4 (2-7)	P < 0.001
Transportation time^d^	23 (17-32)	21 (16-30)	P < 0.001

Emergency cases in farmland/agricultural areas were less frequently transported to high-class emergency hospitals than those in other outdoor workplaces (crude OR: 0.83, 95% CI: 0.81-0.85). The proportion of outpatient discharges was lower, whereas the proportions of outpatient deaths (crude OR: 2.37, 95% CI: 2.24-2.52) and OHCA (crude OR: 1.92, 95% CI: 1.83-2.02) were much higher in farmlands than in other outdoor workplaces (Table 3).

**Table 3:** Comparisons of transportation and severities between farmland and other outdoor workplaces OR: odds ratio, CI: confidential interval, EMS: emergency medical service, IQR: interquartile range ^a^P value by the Wilcoxon/Kruskal-Wallis test is shown for continuous variables. ^b^Emergency medical care centers and emergency hospitals providing advanced patient care.

	Out-of-office workplaces		OR (95% CI) for farmland (all other outdoor workplaces = 1) or P value^a^
	Farmland (N = 72,162)	Other outdoor workplaces (N = 105,761)	
Hospital transportation, number (%)			
High class^b^	16,294 (22.6%)	27,435 (25.9%)	0.83 (0.81-0.85)
Outside the jurisdiction of an ambulance station	16,853 (23.4%)	23,912 (22.6%)	1.04 (1.02-1.07)
Severities, number (%)			P < 0.001
Outpatient discharge	29,249 (40.5%)	48,066 (45.5%)	0.81 (0.80-0.83)
Hospital admission	40,130 (55.6%)	55 938 (52.9%)	1.12 (1.10-1.14)
Outpatient death	2,783 (3.9%)	1,757 (1.1%)	2.37 (2.24-2.52)
OHCA, number (%)	3,710 (5.1%)	2,900 (2.7%)	1.92 (1.83-2.02)

Notably, OHCA accounted for 93.5% (2,620/2,783) and 88.5% (1,554/1,757) of outpatient deaths in emergency cases transported from farmland/agricultural areas and other outdoor workplaces, respectively. This proportion of OHCA was greater in farmland than in other outdoor workplaces (crude OR: 1.88, 95% CI: 1.52-2.32).

Interaction between EMS rurality and farmlands/agricultural lands

Multivariate interaction tests were conducted to determine whether differences in the major characteristics of emergencies between farmland/agricultural areas and other outdoor workplaces were modified by EMS rurality (Table 4).

**Table 4:** Differences in the characteristics of emergency transportation between farmlands/agricultural lands and other outdoor workplaces in rural and non-rural EMS OR: odds ratio, CI: confidential interval, EMS: emergency medical service, IQR: interquartile range ^a^P value by the Wilcoxon/Kruskal-Wallis test is shown for continuous variables. ^b^Interval between emergency call receipt and EMS contact with patients. ^c^Interval between EMS contact with patients and patient accommodation in the ambulance. ^d^Interval between patient accommodation in the ambulance and that in the hospital. ^e^Emergency medical care centers and other high-class emergency hospitals providing advanced emergency care.

Characteristics	Rural municipality EMS			Non-rural municipality EMS			P by multivariate interaction test
	Farmlands (N = 22,624)	Other outdoor workplaces (N = 16,603)	Crude OR (95% CI) (other outdoor workplaces = 1) or P value^a^	Farmlands (N = 49,538)	Other outdoor workplaces (N = 89,158)	Crude OR (95% CI) (other outdoor workplaces = 1) or P value^a^	
2020/2021, number (%)	6,913 (30.6%)	5,273 (31.8%)	0.95 (0.91-0.99)	15,748 (31.8%)	29,059 (32.6%)	0.96 (0.94-0.99)	0.896
Winter, number (%)	2,625 (11.6%)	3,345 (20.2%)	0.52 (0.49-0.55)	6,702 (13.5%)	17,472 (19.6%)	0.64 (0.62-0.66)	<0.001
Daytime, number (%)	18,861 (83.4%)	14,876 (89.6%)	0.58 (0.55-0.62)	41,254 (83.3%)	77,134 (86.5%)	0.78 (0.75-0.80)	<0.001
Age ≥ 60 years, number (%)	19,386 (85.7%)	6,756 (40.7%)	8.73 (8.31-9.16)	42,582 (85.1%)	29,146 (32.7%)	12.6 (12.2-13.0)	<0.001
Male, number (%)	14,890 (65.7%)	15,457 (93.1%)	0.14 (0.13-0.15)	33,870 (68.4%)	84,096 (94.3%)	0.13 (0.13-0.13)	0.002
Medical, number (%)	8,969 (39.6%)	4,729 (28.5%)	1.57 (1.51-1.64)	21,081 (42.6%)	29,616 (33.2%)	1.47 (1.44-1.50)	<0.001
Cardiovascular disease, number (%)	2,815 (12.4%)	1,530 (9.2%)	1.65 (1.58-1.7)	6,104 (12.3%)	7,406 (8.3%)	1.49 (1.47-1.52)	<0.001
EMS time interval, minutes, median (IQR)							
Response time interval^b^	12 (9-16)	11 (8-15)	P < 0.001	11 (8-14)	10 (8-12)	P < 0.001	<0.001
Scene time^c^	6 (3-9)	5 (3-6)	P < 0.001	5 (3-8)	4 (2-6)	P < 0.001	<0.001
Transportation time^d^	24 (17-35)	23 (16-34)	P < 0.001	23 (17-32)	21 (16-29)	P < 0.001	<0.001
Hospital transportation, number (%)							
High class^e^	3,764 (16.6%)	3,375 (20.3%)	0.78 (0.74-0.82)	12,530 (25.3%)	24,060 (27.0%)	0.91 (0.89-0.94)	<0.001
Outside the jurisdiction of an ambulance station	6,199 (27.4%)	4,795 (28.9%)	0.93 (0.89-0.97)	10,654 (21.5%)	19,117 (21.4%)	0.99 (0.97-1.02)	0.018

Rural EMS strengthened the associations of farmlands/agricultural areas with lower incidences in winter and during the daytime and higher proportions of medical and cardiovascular emergencies. The association of farmlands/agricultural areas with a higher proportion of older (≥60 years) patients was stronger in non-rural EMS.

Factors associated with outpatient death in outdoor workplace emergencies by multivariate logistic regression analyses

**Figure 3:**
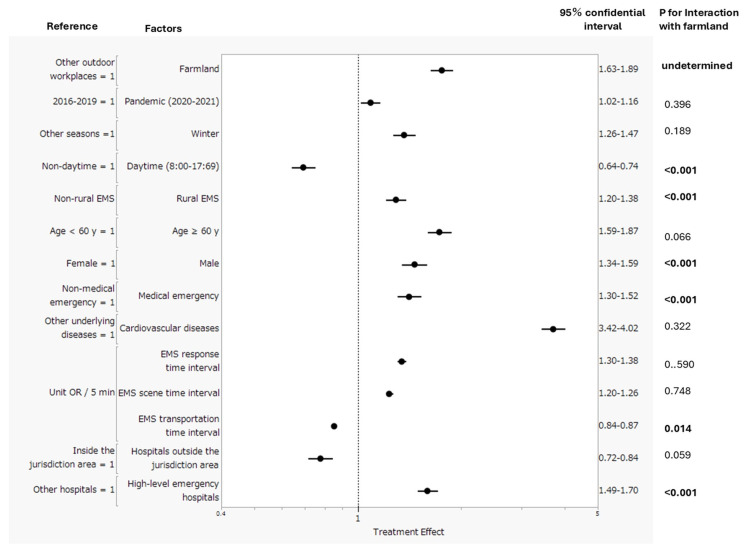
Factors associated with outpatient death in outdoor workplaces EMS: emergency medical service The interaction of each factor and farmland was included in the multivariate regression analysis.

In multivariate logistic regression analysis including major characteristics of emergencies listed in Table 1 and Table 2, all characteristics, in addition to farmlands/agricultural areas, were likely to be associated with outpatient death. Coronavirus disease 2019 pandemic period (2020-2021) (adjusted OR: 1.09, 95% CI: 1.02-1.16), winter (adjusted OR: 1.36, 95% CI: 1.26-1.47), rural EMS (adjusted OR: 1.29, 95% CI: 1.20-1.38), older adult (≥60 years) (adjusted OR: 1.73, 95% CI: 1.59-1.87), male (adjusted OR: 1.46, 95% CI: 1.34-1.59), medical emergencies (adjusted OR: 1.41, 95% CI: 1.30-1.52), cardiovascular diseases (adjusted OR: 3.71, 95% CI: 3.42-4.02), transportation to high-level emergency hospitals (adjusted OR: 1.59, 95% CI: 1.49-1.70), longer EMS response and scene time intervals (unit OR/minute: 1.34, 95% CI: 1.30-1.38 and unit OR/minute: 1.23, 95% CI: 1.20-1.26, respectively), and farmland (adjusted OR: 1.76, 95% CI: 1.63-1.89) were associated with a higher incidence of OHCA. In contrast, daytime (adjusted OR: 0.69, 95% CI: 0.64-0.74), transportation to hospitals outside the jurisdiction area (adjusted OR: 0.78, 95% CI: 0.72-0.84), and a longer EMS transportation time interval (unit OR/minute: 0.85, 95% CI: 0.84-0.87) were associated with a lower incidence of OHCA (Figure 3).

Multivariate interaction tests disclosed that the association of farmlands/agricultural areas with a higher incidence of outpatient death was weakened by daytime (P < 0.001), rural EMS (P < 0.001), male (P < 0.001), and transportation to high-level emergency hospitals (P < 0.001). In contrast, it was strengthened by medical emergency (P < 0.001). The results of these subgroup analyses are summarized in Table 5.

**Table 5:** Subgroups analyses for the difference in the rate of outpatient death between farmlands and other outdoor workplaces EMS: emergency medical service, OR: odds ratio, CI: confidence interval

Subgroups	Outdoor workplaces				OR (95% CI) with other outdoor workplace as reference	
	Farmlands		Others			
	Number	Outpatient death, number (%)	Number	Outpatient death, number (%)	Crude	Adjusted
EMS						
Rural EMS	22,624	928 (4.1%)	16,603	419 (2.5%)	1.65 (1.47-1.86)	1.60 (1.41-1.82)
Non-rural EMS	49,538	1,835 (3.7%)	89,158	1,338 (1.5%)	2.55 (2.38-2.74)	2.51 (2.32-2.71)
Time of day						
Daytime (7:00-18:59)	66,115	2,153 (3.6%)	92,010	1,536 (1.7%)	2.19 (2.05-2.34)	2.07 (1.93-2.23)
Non-daytime	12,047	630 (5.2%)	13,751	221 (1.6%)	3.38 (2.89-3.95)	3.42 (2.89-4.06)
Sex						
Male	48,760	2,077 (4.3%)	99,553	1,717 (1.7%)	2.54 (0.38-2.71)	2.20 (2.06-2.35)
Female	23,402	706 (3.0%)	6,208	40 (0.6%)	4.80 (3.48-6.60)	4.08 (2.95-5.65)
Scene classification						
Medical	30,050	1,870 (6.2%)	34,345	774 (2.3%)	2.88 (2.64-3.13)	2.76 (2.52-3.03)
Non-medical	42,112	913 (2.2%)	71,416	983 (1.4%)	1.59 (1.45-1.74)	1.79 (1.62-1.97)
Emergency hospital						
High class	16,294	767 (4.7%)	27,435	740 (2.7%)	1.78 (1.61-1.98)	1.70 (1.52-1.90)
Other	55,868	2,016 (3.6%)	78,326	1,017 (1.3%)	2.85 (2.64-3.07)	2.53 (0.35-2.78)

Differences in the characteristics of and bystander's response to OHCA between farmlands/agricultural areas and other outdoor workplaces

Compared with other outdoor workplaces, OHCA cases in farmlands/agricultural areas had many disadvantageous characteristics for survival: higher proportions of older patients (≥60 years) and unwitnessed cases, low rate of bystander cardiopulmonary resuscitation (CPR), and low incidences of shockable initial rhythms and public access defibrillation, regardless of EMS rurality (Table 6). None of the variables showed a significant interaction between EMS rurality and farmlands/agricultural lands in the multivariate interaction test.

**Table 6:** Differences in the characteristics of and bystander's response to OHCA between farmlands and other outdoor workplaces in rural and non-rural municipality EMS OR: odds ratio, CI: confidential interval, EMS: emergency medical service, IQR: interquartile range, CPR: cardiopulmonary resuscitation, PAD: public access defibrillation with an automated external defibrillator, OHCA: out-of-hospital cardiac arrest ^a^P value by the Wilcoxon/Kruskal-Wallis test was shown for continuous variables. OR (95% CI) was shown for nominal variables when the chi-squared test detected a significant difference. ^b^Interval between emergency call receipt and EMS contact with patients.

Characteristics of and bystander's response to OHCA	Rural municipality EMS			Non-rural municipality EMS			P by multivariate interaction test
	Farmlands (N = 1,083)	Other outdoor workplaces (N = 540)	Crude OR (95% CI) (other outdoor workplaces = 1) or P value^a^	Farmlands (N = 2,438)	Other outdoor workplaces (N = 2,227)	Crude OR (95% CI) (other outdoor workplaces = 1) or P value^a^	
2020 and 2021	335 (30.9%)	171 (31.7%)	0.97 (0.77-1.21)	826 (33.9%)	798 (35.8%)	0.92 (0.81-1.04)	0.554
Age ≥ 60 years, number (%)	997 (92.1%)	291 (53.9%)	9.92 (7.51-13.1)	2,227 (91.4%)	1,104 (49.6%)	10.7 (9.11-12.6)	0.407
Male, number (%)	801 (78.0%)	521 (96.5%)	0.10 (0.06-0.17)	1,872 (76.8%)	2,188 (98.3%)	0.06 (0.04-0.08)	0.830
Witness status, number (%)			P < 0.001			P < 0.001	0.849
Unwitnessed	846 (78.1%)	241 (44.6%)	4.43 (3.54-5.53)	1,840 (75.5%)	898 (40.3%)	4.52 (3.99-5.13)	Excluded
EMS-witnessed	51 (4.7%)	52 (9.6%)	0.46 (0.31-0.69)	136 (5.6%)	204 (9.2%)	0.59 (0.47-0.73)	Excluded
Family-witnessed	121 (11.2%)	18 (3.3%)	3.65 (2.20-6.05)	247 (10.1%)	50 (2.3%)	4.91 (3.60-6.70)	Excluded
Friend/colleague-witnessed	30 (2.8%)	208 (38.5%)	0.05 (0.03-0.07)	107 (4.4%)	990 (44.5%)	0.06 (0.05-0.07)	Excluded
Other bystanders-witnessed	35 (3.2%)	21 (3.9%)	0.83 (0.48-1.43)	108 (4.4%)	85 (3.8%)	1.17 (0.87-1.56)	Excluded
Bystander CPR, number (%)	465 (42.9%)	278 (51.5%)	0.71 (0.58-0.87)	1,075 (44.1%)	1,107 (49.7%)	0.79 (0.71-0.90)	0.247
PAD, number (%)	4 (0.4%)	15 (2.8%)	0.13 (0.04-0.39)	7 (0.3%)	98 (4.4%)	0.06 (0.03-0.13)	0.248
Of presumed cardiac etiology, number (%)	596 (55.0%)	228 (42.2%)	1.67 (1.36-2.06)	1,075 (44.1%)	1,107 (49.7%)	0.80 (0.71-0.90)	0.430
Medical, number (%)	717 (66.2%)	237 (43.9%)	2.50 (2.03-3.09)	1,727 (70.8%)	1,204 (54.1%)	2.06 (1.83-2.33)	0.937
Shockable initial rhythm, number (%)	65 (7.3%)	94 (21.5%)	0.29 (0.21-0.41)	186 (9.2%)	500 (26.9%)	0.28 (0.23-0.33)	0.801
EMS time interval, minutes, median (IQR)							
Response time interval^b^	13 (10-18)	13 (9-18)	P = 0.126	12 (9-15)	11 (8-14)	P < 0.001	0.380
Scene time interval^c^	7 (4-10)	6 (4-9)	P = 0.100	5 (4-8)	5 (3-8)	P = 0.109	0.768
Transportation time interval^d^	20 (13-29)	19 (12-28)	P = 0.139	19 (13-26)	17 (11-25)	P < 0.001	0.132
EMS transportation, number (%)							
High-class emergency hospital	169 (15.6%)	128 (23.7%)	0.60 (0.46-0.77)	911 (37.4%)	1,189 (53.4%)	0.52 (0.46-0.59)	0.478

Factors associated with a neurologically favorable outcome of OHCA in outdoor workplaces

**Figure 4:**
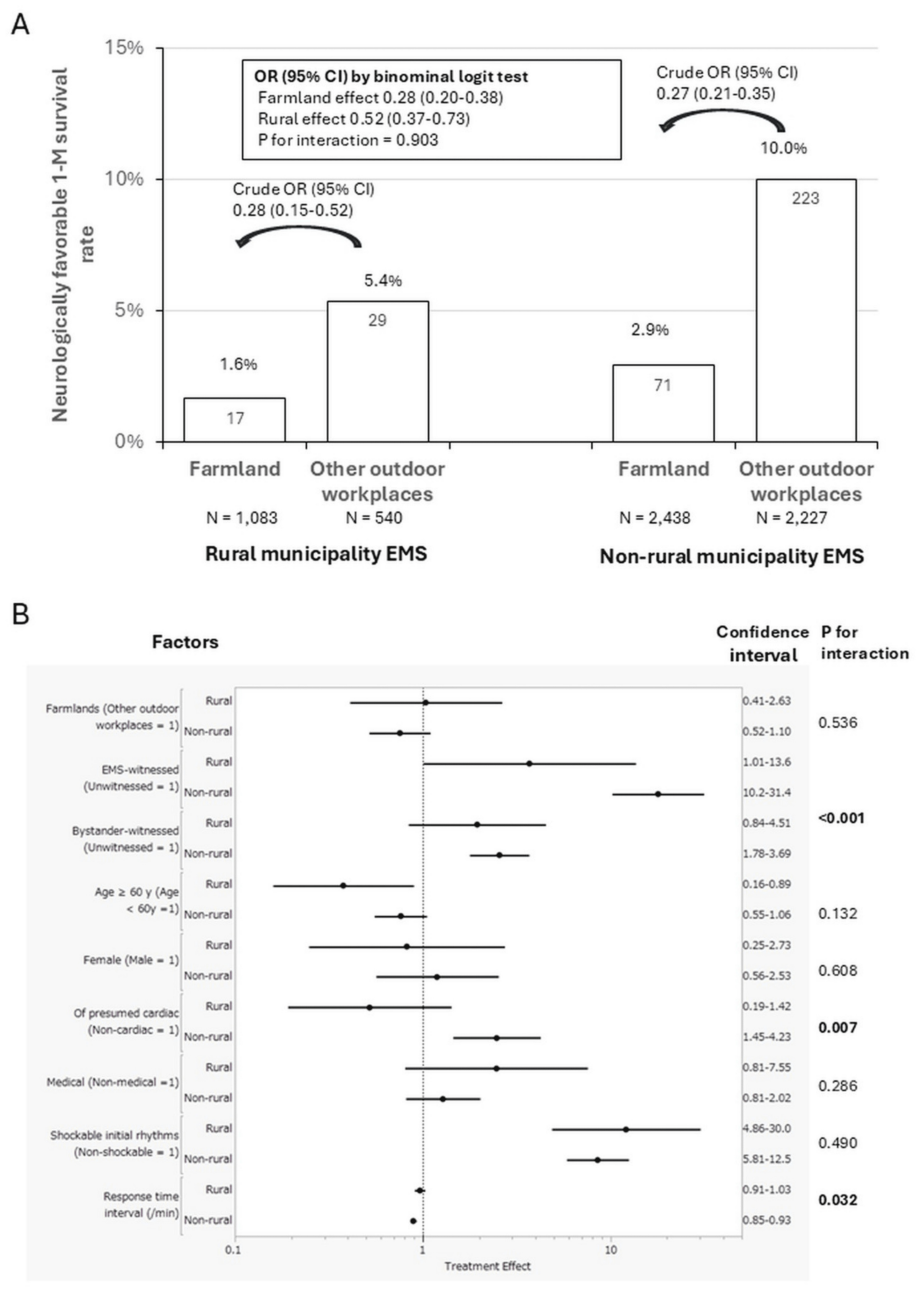
Neurologically favorable one-month survival from OHCA in outdoor workplaces in rural and non-rural EMS EMS: emergency medical service, response time interval: time interval between emergency call receipt and EMS contact to patients, OHCA: out-of-hospital cardiac arrest, OR: odds ratio, CI: confidence interval A: Comparisons in outcomes by binominal interaction test B: Factors associated with neurologically favorable one-month survival of OHCA in outdoor workplaces

The neurologically favorable one-month survival rate of OHCA in farmlands/agricultural areas was as low as 1.6% in rural EMS and 2.9% in non-rural EMS, much lower than that in other outdoor workplaces, regardless of EMS rurality (5.4% and 10%, respectively, in rural and non-rural EMS): crude OR (95% CI) by binominal logit analysis was 0.28 (0.20-0.38) for farmland and 0.52 (0.37-0.73) for rural EMS (P for interaction = 0.903) (Figure 4A). However, when multivariate logistic regression analysis including the characteristics of OHCA listed in Table 4 was applied to determine major factors associated with OHCA outcomes in outdoor workplaces, farmlands/agricultural areas were not significantly associated with the outcome (adjusted OR: 1.04, 95% CI: 0.41-2.63 and adjusted OR: 0.76, 95% CI: 0.52-1.10, respectively, in rural and non-rural EMS). Regardless of EMS rurality, EMS-witnessed OHCA (adjusted OR: 3.70, 95% CI: 1.01-13.6 and adjusted OR: 17.9, 95% CI: 10.2-31.4) and shockable initial rhythms (adjusted OR: 12.1, 95% CI: 4.86-30.0 and adjusted OR: 8.53, 95% CI: 5.81-12.5) were associated with a better outcome, whereas prolonged EMS response time interval (adjusted unit OR/minute: 0.97, 95% CI: 0.91-1.03 and adjusted unit OR/minute: 0.89, 95% CI: 0.85-0.93) was associated with a worse outcome (Figure 4B). Notably, the association of EMS-witnessed OHCA with a better outcome was strengthened in rural EMS (P for interaction < 0.001). In contrast, the association of EMS response time with a worse outcome was strengthened in non-rural EMS (P for interaction = 0.032).

## Discussion

As expected from the previous reports [3,4] and guidelines [2], the present study shows that the incidence of medical and severe emergencies was higher in farmlands and agricultural areas. Furthermore, regardless of the EMS rurality, the outcomes of OHCA in farmlands were worse than those in other outdoor workplaces owing to low proportions of witnessed cases, cases involving younger patients, and low incidences of bystander CPR, shockable initial rhythm, and PAD usage. The lack of termination of resuscitation in Japan also contributes to the very low rate of survival from OHCA. These features of medical emergencies and OHCA are mainly attributable to the increasingly aging population and rapidly declining number of farmers in Japan [15].

In this study, more than 50% of patients transported from farmlands or agricultural areas were 75 years or older, whereas only 5% of those transported from other outdoor workplaces were in the same age group. This indicates that farmlands and agricultural areas are rural locations where older workers are prone to emergencies [11]. Additionally, the proportion of older farmers (agricultural holders) is growing globally, whereas the proportion of younger farmers is decreasing, particularly in Asia [23]. Therefore, managing medical emergencies among elderly farmers is a pressing global concern that requires immediate attention and improvement.

A higher proportion of outpatient death in emergencies in farmlands and agricultural areas than in other outdoor workplaces is not merely attributed to aging farmers, as farmlands/agricultural areas were associated with a higher proportion of outpatient death even after multivariate logistic regression, including age, was applied. The Occupational Health and Safety Law mandates annual health checkups and medical examinations in Japan. However, the National Survey of Living Conditions [24] showed that the percentage of farmers receiving health and/or medical examinations is as low as 63% in Japan, lower than in other countries (70%), presumably because of their low interest in their health [25]. Furthermore, concerns about contracting the new coronavirus strain continue to dissuade people, including farmers, from undergoing medical checkups. This might have led to a weak but significant association of the two-year-long pandemic with a high incidence of outpatient death in outdoor workplaces [18].

Delays in detecting emergency disease outbreaks and activating local EMS systems are another serious problem in farmlands/agricultural areas [6,11]. These delays were likely to cause a higher proportion of outpatient deaths in farmlands/agricultural areas as the proportions of OHCA in outpatient deaths and unwitnessed cases in OHCA were higher in farmlands/agricultural areas than in other outdoor workplaces. Furthermore, the association of farmlands with higher proportions of older patients and medical and severe emergencies was significantly stronger in non-rural municipality EMS. A recent survey of rural communities in Japan emphasized the importance of adaptable and robust support systems that address various health issues, including establishing collaborative relationships between rural communities and medical institutions [25].

Except for metropolitan regions, non-rural municipalities in Japan are composed of expanded residential areas, where most medical facilities and EMS ambulance stations are located, and surrounding non-residential areas, where farmlands/agricultural areas exist. Owing to aging and depopulation in Japan, elderly farm households lack sufficient labor and rely on mechanization, whereas younger generations have lost interest in agriculture. Consequently, many farm households, particularly in non-rural municipalities, are transitioning from commercial to self-sufficient or private businesses, leaving the agricultural community for commercial farming, which may help organize a health management system and cooperative agricultural work. Agriculture is a major industry in rural municipalities, and large agricultural cooperatives are forming in some municipalities. Thus, in non-rural municipalities, health risk management of farmlands/agricultural areas, including early detection of emergencies, is much poorer than that of other outdoor workplaces owned and managed by business entities [11,25].

The outcomes of OHCA cases in workplaces other than farmlands/agricultural areas have been reported to be better than those in other locations [8-10], presumably because of the high density of people and AED implementation with workplace training. This study showed that farmlands/agricultural areas were associated with poorer bystander and EMS responses to OHCA. The lower neurological survival rate in farmlands/agricultural areas than in other outdoor workplaces can be attributed to the high proportion of older patients, low chance of witnessing, low incidence of bystander CPR provision and PAD usage, and delayed EMS arrival at the scene. This assumption was supported by the lack of significant differences in neurologically favorable outcomes between farmlands/agricultural areas and other outdoor workplaces in multivariate logistic regression analyses, including bystander and EMS responses to OHCA (Figure 2B).

As expected, the response time interval, defined as the interval between emergency call receipt and EMS contact with the patient, was largely prolonged in farmlands/agricultural areas. Prolongation of this critical time interval might be associated with the severity of diseases and worse prognosis. This time interval comprises three major time interval components. The time interval between the receipt of an emergency call and EMS vehicle dispatch, the first component, was more frequently prolonged in farmlands than in other outdoor workplaces. This is presumably due to the difficulty in obtaining exact location information. The time interval between EMS vehicle dispatch and arrival near the scene, the second component, mainly depends on road conditions and the distance between the EMS station and the scene, which might be prolonged in rural farmlands. The time interval between EMS vehicle arrival near the scene and EMS contact to the patient might be extended because of the narrow field pathway for an emergency vehicle [7].

Considering the aging society, preventive and educational approaches for farmers are needed to decrease severe medical emergencies and improve the outcomes of OHCA in farmlands. Preventive strategies should include restructuring agricultural communities to enable community-based health management, round-the-clock medical examinations, and complementary or cooperative work, which can augment the probability of witnessing OHCA [25]. This study showed that rural municipalities were associated with poorer outcomes of outdoor OHCA, regardless of farmland or other outdoor workplaces. Effective and simplified basic life support and first-aid training should be provided to outdoor workers, particularly older workers [17,26]. The training course should include learning prodrome symptoms and recognizing the importance of early access to the EMS system (emergency call).

Therefore, the mechanization and automation of agriculture should include modern lifesaving mobile network systems [27,28]. The increasing size of farmland in recent years should be accompanied by improved emergency detection and accessibility. The EMS and agricultural communities should organize a dispatch and delivery system for well-trained first responders and portable AEDs [29,30]. Preventing and managing health crises is an urgent issue that should be included in the Sustainable Development Goals (SDGs) for agriculture.

This study had some limitations inherent to its observational design. First, the relationships between workplaces and workers cannot be entirely established. It is possible that OHCA cases among nonworkers on farmlands were also included. Second, data based on reports from bystanders before the arrival of the EMS could be inaccurate. Third, the lack of established regulations recommending the termination of resuscitation in prehospital settings means that data collected from other countries may yield different results. Additionally, bystander CPR training experience and quality were not examined, which could have influenced the association between bystander CPR and favorable outcomes. Finally, lack of data completeness, low validity, and ascertainment bias are potential limitations similar to those in most epidemiological studies. However, to the best of our knowledge, this is the first nationwide study to investigate medical emergencies and OHCA in agricultural settings and addresses an issue that should be of great interest to the international community.

## Conclusions

Regardless of EMS rurality, the incidence of medical and major emergencies is higher in farmlands/agricultural areas than in other outdoor workplaces. EMS time intervals, including response, scene, and transport time intervals, are longer in farmlands/agricultural areas. The association of farmlands/agricultural areas with a higher incidence of outpatient deaths is weakened in non-rural EMS. Detection of serious emergencies is likely to be very poor in farmlands/agricultural areas. Bystander and EMS responses to OHCA were more likely to be inadequate, and consequently, the outcomes of OHCA were worse in farmlands/agricultural areas. The prevention and management of health crises is an urgent issue that should be included in the SDGs for agriculture.
